# Knowledge of Sexually Transmitted Infections and Its Association With Condom Use Self-Efficacy (CUSES) Among People Living With HIV (PLHIV) in Public Health Clinics in Selangor, Malaysia

**DOI:** 10.7759/cureus.68154

**Published:** 2024-08-29

**Authors:** Syameme Padzel, Farnaza Ariffin, Salma Yasmin Mohd Yusuf, Mariam Mohamad

**Affiliations:** 1 Primary Care Medicine, Faculty of Medicine UiTM, Selangor, MYS; 2 Public Health Medicine, Faculty of Medicine UiTM, Selangor, MYS

**Keywords:** primary care, sexual behavior, sti knowledge, plhiv, condom use self-efficacy

## Abstract

Background

Condom use self-efficacy is critical in preventing the spread of HIV among people living with HIV (PLHIV). Knowledge of sexually transmitted infections (STIs) is a crucial factor in promoting safer sexual behaviors. However, there is scarce data on its association with condom use self-efficacy, particularly among PLHIV.

Objectives

This study aims to determine the association between knowledge of STIs and other demographic and behavioral factors with condom use self-efficacy among PLHIV in public health clinics at Hulu Langat, Selangor.

Methods

A cross-sectional study involved PLHIV attending public health clinics in Selangor, Malaysia. Participants were recruited using convenience sampling. Eligibility criteria included being 18 years or older, able to read and comprehend the Malay language, and having not been diagnosed with psychiatric illness or learning difficulties that may impede their ability to answer the questionnaire. Data were collected from December 2023 to March 2024 through self-administered questionnaires assessing demographic characteristics, sexual practice, clinical factors, knowledge of STIs, and condom use self-efficacy. Multiple linear regressions were performed to identify factors associated with condom use self-efficacy.

Results

The mean score for condom use self-efficacy was 77.72 ± 12.88, and the mean score for knowledge of STIs was 30.87 ± 5.50. The higher STI knowledge score was associated with higher condom use self-efficacy (B = 0.73, p < 0.001). Other significant factors were higher household income (B = 9.27, p < 0.001), recent sexual activity within the last three months (B = -4.34, p < 0.001), engaging in receptive anal sex (B = 7.06, p < 0.001), and not using a condom during the last sexual intercourse (B = -4.43, p < 0.001) were associated with condom use self-efficacy.

Conclusion

The study confirms that good STI knowledge increases condom use self-efficacy among PLHIV, therefore highlighting the need to educate those at risk with better knowledge of STIs. It also highlights the importance of conducting interventions for those at higher risk due to lower condom use self-efficacy and exploring their barriers towards the use of condoms.

## Introduction

HIV infection still poses a great challenge in curbing and controlling the spread of the virus. According to the AIDS Monitoring Report, Malaysia recorded 86,142 people living with HIV (PLHIV), with an annual incidence of 3,177 by the end of 2022. The state of Selangor exhibited the highest concentration of cases, representing 31% of the national total, with 966 new infections, 135 AIDS cases, and 145 deaths within one year [1]. A report from the UNAIDS data shows that the landscape of HIV transmission in some countries, such as Malaysia, has shifted from people who inject drugs (PWID) to sexual transmission as the predominant mode of spreading the virus [2]. This transition underscores the critical need to enhance prevention measures to curb HIV transmission effectively.

Condom use has been identified as one of the most effective means of preventing the spread of HIV [3,4]. Despite being a highly effective preventive measure, the use of condoms is often hindered by a lack of public education, cultural and religious beliefs, and economic constraints, which contribute to the ongoing transmission of HIV [5,6]. The consistent use of condoms among PLHIVs in many studies is less than 50%, highlighting the need for targeted interventions to address this issue [7-9]. The HIV epidemic in Malaysia is uniquely concentrated among four key populations (KPs), and they are people who inject drugs (PWID), female sex workers (FSW), transgender (TG), and men who have sex with men (MSM). The available data on condom use among these KPs are 47.5% among heterosexual partners to PWID [7], 75% usage in MSM, 41.8% usage in TG, and 40.8% usage in FSW, which are considerably low and put these populations at high risk [10]. 

“Condom use self-efficacy" is defined as an individual's confidence in their ability to use a condom correctly [11] and is directly linked to successful condom use [12]. Higher self-efficacy leads to greater intentions and frequency of condom use, promoting safer sexual practices [13]. Low self-efficacy is found to lead to inconsistent condom use, thus increasing the risk of transmission [5,14]. The concept of ‘self-efficacy’ by Bandura refers to an individual’s belief in their ability to execute behaviors necessary to produce specific performance attainments [15].

Knowledge of sexually transmitted infections (STI) is linked to condom use [8,16], yet STI knowledge remains low among key populations [17]. In Malaysia, there was a study on the level of STI knowledge but not among PLHIV [18,19]. Globally, there were studies on factors influencing self-efficacy in condom use, especially in high-risk groups [20,21], but not on the relationship between knowledge of STI and condom use self-efficacy among PLHIV. This study aims to determine the association between the knowledge of STIs and other factors associated with condom use self-efficacy among PLHIV in Selangor, Malaysia. Identifying the associated factors may assist in determining those who require intervention to aid in reducing HIV transmission.

## Materials and methods

Study design and population

A cross-sectional study was conducted among adult HIV patients who attended three public health clinics in Selangor, Malaysia, using a self-administered questionnaire (Appendix). The data collection period was from December 2023 until March 2024. The participants recruited were PLHIV who were adults (18 years and older) and able to read and comprehend the Malay language. They may have been diagnosed with HIV at any time before the data collection. Those who have been diagnosed with a psychiatric illness or learning difficulties that impede their ability to complete the questionnaire were excluded from the study. Sample size calculation.

Sample size calculation

The sample size estimation was based on a study by Rahim et al., which indicated that 53% of PLHIV had high condom use self-efficacy scores. Using this prevalence, the formula estimated a required sample size of 369 with a significance level of 0.05 and a power of 80%. Considering a 20% non-response rate, the final calculated sample size was adjusted to 442.

Sampling method and data collection

The sample population included all PLHIV who attended the selected public health clinics during the data collection period. Participants were recruited using convenience sampling from PLHIV attending the three public health clinics with a dedicated HIV clinic. The researcher approached potential participants in the waiting area, introduced the study, and invited interested PLHIV to participate. Those who agreed were taken to a separate room, screened for eligibility, and provided with the study information sheets and consent forms. All participants were given time to read through the study information sheets, sign the consent form, and complete the self-administered questionnaire. This questionnaire consisted of sociodemographics, clinical factors, knowledge of STIs, and condom use self-efficacy. Knowledge of STIs was measured using a validated tool in the Malay language, and the condom-use self-efficacy scale (CUSES) Malay version was used to measure condom-use self-efficacy. They were assured of anonymity and confidentiality and that their responses would not affect their clinical care. Participants filled out the questionnaires in a separate area and were given pens and clipboards. The researcher was available to answer questions during data collection. After completing the questionnaires, participants returned them to the researcher and received pamphlets on safe sexual practices.

Study tool

The questionnaire consists of five sections. Section A consists of sociodemographic data comprising factors such as gender, date of birth, age, ethnicity, marital status, level of education, religion, and employment status. Section B consisted of questions on the sexual practices of the participants, and Section C contained questions on clinical characteristics such as treatment status and viral suppression. 

Section D is the validated Malay questionnaire that measures the participants’ knowledge of STI. The questionnaire was developed in the Malay language by Mansor et al., and it has good internal consistency (Cronbach alpha of 0.878) [22]. The questionnaire has five domains, which are knowledge of the type of STI (10 items), mode of STI transmission (seven items), risk factors for STI (seven items), signs and symptoms of STI (eight items), and control and preventive measures of STI (six items), for a total of 38 questions. Each item has three option answers: 'yes,' 'no,' or 'don't know'. A correct answer receives one point, while an incorrect answer or ‘don’t know answer receives zero points. The highest possible score is 38, and the lowest score is 0. A higher score indicates better knowledge of STIs. 

Section E is the Malay version of the 'Condom Use Self-Efficacy Scale', also known as CUSES, that consists of 27 self-administered items. The original English version was developed by Brafford and Beck in 1991 to assess college students’ conviction in their ability to properly apply and negotiate the use of a condom during sexual activity [23]. It has been translated and validated among the Malaysian population. The Malay CUSES went through a forward and backward translation, content validation, and psychometric analysis [23]. It has good overall internal consistency (Cronbach alpha of 0.878) (23). The items are measured using a five-point Likert scale ranging from strongly disagree (0 points) to strongly agree (4 points). Higher scores indicate greater condom self-efficacy. The Malay CUSES questionnaire maintained the original four subscales, which are mechanics (10 items), perceived barriers (seven items), assertiveness (seven items), and intoxicants (three items). Each subscale has its own score based on the number of questions it contains. The authors' consent for both the knowledge of the STI questionnaire and CUSES-M was obtained before the conduct of the study.

Statistical analysis

IBM Corp. Released 2021. IBM SPSS Statistics for Windows, Version 28.0. Armonk, NY: IBM Corp. was used for data entry and statistical analysis. Descriptive statistics such as mean, standard deviation (SD), frequency, and percentage were used to summarize sociodemographic, clinical, and sexual practices as well as the score of STI knowledge and condom use self-efficacy.

We conducted both simple and multiple linear regression analyses to examine the factors associated with condom use self-efficacy. Variables with a p-value less than 0.05 in the simple linear regression were included in the stepwise multiple linear regression model. The stepwise method was preferred for its ability to iteratively add and remove variables based on significance, resulting in a more efficient and parsimonious model with improved predictive accuracy and reduced multicollinearity. This approach balances the strengths of both forward selection and backward elimination while avoiding the drawbacks of including non-significant variables, as seen in the standard enter method. The analysis detected multicollinearity between the variables 'sexual orientation’ and ‘type of sexual practice’ as shown by its high variance inflation factor (VIF) > 10. To address this issue, the variable ‘sexual orientation’ was removed from the final model as it was deemed less theoretically important. To ensure the validity of the multiple linear regression model, several assumptions were checked. Scatterplots and residual plots used confirmed the linearity between the dependent and independent variables. The Durbin-Watson value was 1.59, indicating the independence of the residuals. There was homoscedasticity, as assessed by visual inspection of a plot of standardized predicted values. The residuals were normally distributed, as evidenced by the bell-shaped histogram. The statistical significance level was set at a p-value less than 0.05.

Ethical consideration

The study was conducted following the ethical principles outlined in the Declaration of Helsinki. Ethical approval was obtained from the Ethics Committee of the Faculty of Medicine, University Teknologi MARA (UITM), and the Malaysia Research Ethical Committee (MREC). Informed consent was secured from all participants before their inclusion in the study. Participants were provided with comprehensive information about the study's purpose, procedures, potential risks, and benefits. They were assured of their right to withdraw from the study at any time without any consequences to their medical treatment. The anonymity and confidentiality of the participant's data were strictly maintained throughout the study. Data was securely stored, and only the research team had access to it.

## Results

A total of 375 PLHIV were approached. Three of the PLHIV refused to participate in the study, yielding 84% of the response rate. All 372 of the participants fulfilled the eligibility criteria, completed the questionnaire, and were included in the final analysis. The average age of the participants was 38.23 ± 11.30 years. As shown in Table 1, 326 (87.6%) of the participants were male. Most of the participants earned low to middle income, which were 173 (46.5%) and 176 (47.3%), respectively, while only 19 (5.1%) of the participants were from the high-income group. Sexual behavior data indicated that 228 (61.3%) of participants had been sexually active within the last three months. Half of the participants identified were homosexual, 186 (50.0%). Most were on anti-retroviral (ARV) treatment; 366 (98.4%), and 301 (80.9%) had achieved viral suppression.

**Table 1:** Characteristic of adult PLHIV in Selangor, Malaysia (n=372) The data has been presented as n (%) for categorical variables and mean ± SD for continuous variables. ARV: Antiretroviral, CI: Confidence interval, CUSES: Condom use self-efficacy, HIV: Human immunodeficiency virus, PLHIV: People living with HIV, SD: Standard deviation

Variables		Frequency, n (%)	Mean ± SD	Minimum score	Maximum score
Age (years)			38.23 ± 11.30	19	70
Gender	Male	326 (87.6)			
	Female	46 (12.4)			
Ethnicity	Malay	242 (65.1)			
	Chinese	93 (25.0)			
	Indian	32 (8.6)			
	Others	5 (1.3)			
Marital status	Single	277 (74.7)			
	Married	50 (13.5)			
	Widow/divorcee	44 (11.9)			
Education level	No formal education	2 (0.5)			
	Primary	10 (2.7)			
	Secondary	152 (40.9)			
	Pre-university	13 (3.5)			
	Tertiary	195 (52.4)			
Religion	Muslim	243 (65.3)			
	Christian	28 (7.5)			
	Buddha	78 (21.0)			
	Hindu	23 (6.2)			
Occupation	Government employee	17 (4.6)			
	Private employee	218 (61.3)			
	Self-employed	88 (23.7)			
	Housewife/unemployed	46 (12.4)			
	Others	2 (0.5)			
	Missing	1 (0.3)			
Household income	RM4849 and below	173 (46.5)			
	RM4850 – RM10959	176 (47.3)			
	RM10960 and above	19 (5.1)			
	Missing	4 (1.1)			
Last sexual activity	Less than 3 months	228 (61.3)			
	3 months or more	115 (30.9)			
	Not sure	29 (7.8)			
Sexual orientation	Heterosexual	117 (31.5)			
	Homosexual	186 (50.0)			
	Bisexual	59 (15.9)			
	Not sure	10 (2.7)			
Type of sexual practice	Vaginal sex	117 (31.5)			
	Oral sex	12 (3.2)			
	Receptive anal sex	29 (7.8)			
	Insertive anal sex	39 (10.5)			
	Multiple sexual practices	166 (44.6)			
	Missing	9 (2.4)			
Multiple sexual partner	Yes	199 (53.5)			
	No	162 (43.5)			
	Missing	11 (3.0)			
Age of sex debut (years)			19.52 ± 3.00	13	31
Condom use during 1^st^ sexual intercourse	Yes	52 (14.0)			
	No	293 (78.8)			
	Non-disclosure	27 (7.3)			
Condom use during last sexual intercourse	Yes	255 (68.5)			
	No	86 (23.1)			
	Non-disclosure	31 (8.3)			
Type of visit to health clinic	New visit	4 (1.1)			
	Follow up	368 (98.9)			
Duration of HIV (years)			6.61 ± 5.17	1	28
Duration of follow up (years)			3.95 ± 3.20	1	21
ARV treatment status	Yes	366 (98.4)			
	No	4 (1.1)			
Duration of ARV treatment			5.98 ± 4.58	1	27
Awareness of partners’ HIV status	Yes	286 (76.9)			
	No	82 (22.0)			
	Missing	4 (1.1)			
Viral suppressed status (self-reported)	Yes	301 (80.9)			
	No/unsure	71 (19.1)			
Alcohol consumption	Yes	182 (48.9)			
	No	190 (51.1)			
Illicit drug consumption	Yes	106 (28.5)			
	No	266 (71.5)			
Smoking status	Current/daily smoker	104 (28.0)			
	Current/occasional smoker	12 (3.2)			
	Nonsmoker	230 (61.8)			
	Missing	26 (7.0)			

Table 2 describes the participants’ scores of STI knowledge. Overall, the mean score of STI knowledge was 30.87 ± 5.50, representing 81.2% of the maximum possible score. The "risk factors of STI" and the ‘preventive measures of STI’ subdomains scored the highest, with a mean equating to 95.1% and 93.3% of the maximum score, respectively. The "Transmission of STI" subdomain also showed a high level of knowledge, with a mean score of 6.46 ± 1.13, accounting for 92.2% of the maximum score. In contrast, the "Types of STI" subdomain had the lowest mean score at 6.73 ± 2.35, representing 67.3% of the maximum score. Lastly, the "Symptom of STI" subdomain had a mean score of 5.42 ± 1.82, or 67.7% of the maximum score.

**Table 2:** Knowledge of STI score of the participants The data has been presented as mean ± SD SD: Standard deviation, STI: Sexually transmitted infection

Variables		Mean SD	Percentage from maximum score (%)	Minimum score	Maximum score
Total score Knowledge of STI		30.87 ± 5.50	81.2	5	38
Subdomain score	Types of STI (0-10)	6.73 ± 2.35	67.3	0	10
	Risk factors of STI (0-7)	6.46 ± 1.13	92.2	0	7
	Transmission of STI (0-7)	6.66 ± 1.00	95.1	0	7
	Symptom of STI (0-8)	5.42 ± 1.82	67.7	0	8
	Preventive measures of STI (0-6)	5.60 ± 0.74	93.3	1	6

Table 3 shows the CUSES score and its subdomains. The analysis revealed a mean total score of 77.72 ± 12.88, corresponding to 72.0% of the maximum possible score. Among the subdomains, "Mechanics" had the highest mean score at 30.03 ± 5.09, which is 75.1% of the maximum score. The "Assertiveness" subdomain followed closely with a mean score of 20.77 ± 3.94, representing 74.2% of the maximum score. The "Perceived barrier" subdomain had a mean score of 19.46 ± 4.85, accounting for 69.5% of the maximum score, while the "Intoxicant" subdomain scored the lowest at 7.46 ± 1.95, or 62.2% of the maximum score. 

**Table 3:** CUSES score of the participants The data has been presented as mean ± SD CUSES: Condom use self-efficacy scale, SD: Standard deviation

Variables		Mean ± SD	Percentage from maximum score (%)	Minimum score	Maximum score
Total score of CUSES (0-108)		77.72 ± 12.88	72.0	25	108
Subdomain score	Mechanics (0-40)	30.03 ± 5.09	75.1	0	40
	Assertiveness (0-28)	20.77 ± 3.94	74.2	0	28
	Perceived barrier (0-28)	19.46 ± 4.85	69.5	7	28
	Intoxicant (0-12)	7.46 ± 1.95	62.2	0	12

Table 4 provides a detailed cross-tabulation of condom use self-efficacy scores across various demographic and other factors and the simple linear regression analysis to determine factors associated with condom use self-efficacy. In the simple linear regression analysis, several factors were significantly associated with condom use self-efficacy, including gender, marital status, education level, religion, occupation, household income, sexual activity, sexual orientation, type of sexual practice, multiple sexual partners, age of sex debut, condom use during last sexual intercourse, duration of HIV, duration of ARV treatment, awareness of partner’s HIV status, viral suppressed status, illicit drug consumption, and knowledge of STI.

**Table 4:** Cross-tabulation and simple linear regression of the factors associated with condom use self-efficacy of the participants The cross-tabulation data has been presented as mean ± SD. Simple linear regression has been presented as B (95% CI). The p-value is considered statistically significant at p-value < 0.05. Dependent variable: condom use self-efficacy. ARV: Antiretroviral, B: Regression coefficient, CI: Confidence interval, CUSES: Condom use self-efficacy, HIV: Human immunodeficiency virus, SD: Standard deviation

Variables		Mean CUSES score ± SD	Simple linear regression	
			B (95% CI)	p-value
Age (years)			0.59 (-0.199, 0.033)	0.161
Gender	Male	78.45 ± 12.18	Ref	
	Female	72.52 ± 16.29	-5.93 (-9.88, -1.98)	0.003
Ethnicity	Chinese	77.24 ± 13.49	Ref	
	Malay	78.53 ± 12.76	1.28 (-1.79, 4.35)	0.411
	Indian	72.16 ± 16.36	-5.08 (-10.24, 0.77)	0.053
	Others	83.40 ± 11.68	6.16 (-5.39, 17.72)	0.295
Marital status	Single	78.45 ± 12.76	Ref	
	Married	80.74 ± 10.41	2.29 (-1.51, 6.09)	0.236
	Widow/divorcee	69.77 ± 13.62	-8.675 (-12.69, -4.66)	0.001
Education level	Secondary education	74.90 ± 11.90	Ref	
	No formal	61.00 ± 28.29	-13.90 (-31.48, 3.68)	0.121
	Primary	71.40 ± 14.27	-3.50 (-11.57, 4.56)	0.394
	Pre-university	76.46 ± 4.81	1.56 (-5.58, 8.70)	0.668
	Tertiary	80.49 ± 13.17	5.59 (2.92, 8.26)	<0.001
Religion	Muslim	78.61 ± 12.05	Ref	
	Christian	80.46 ±15.85	1.85 (-3.16, 6.86)	0.468
	Buddha	75.83 ± 13.60	-2.78 (-6.05, 0.49)	0.095
	Hindu	71.30 ± 13.17	-7.31 (-12.79, -1.83)	0.009
Occupation	Private employee	80.76 ± 10.35	Ref	
	Government employee	79.71 ± 16.09	-1.05 ( -7.00, 4.90)	0.728
	Self-employed	76.07 ± 12.35	-4.69 (-7.67, -1.71)	0.002
	Housewife/unemployed	66.35 ± 16.25	-14.41 (-18.24, -10.57)	<0.001
	Others	62.00 ± 16.97	-18.76 (-35.54, -1.98)	0.029
Household income	RM4850 – RM10959	79.66 ± 10.70	Ref	
	RM4849 and below	74.69 ± 13.41	-4.98 (-7.52, -2.44)	<0.001
	RM10960 and above	90.89 ± 10.75	11.23 (5.51, 16.96)	<0.001
Last sexual activity	Less than 3 months	81.18 ± 11.35	Ref	Ref
	3 months or more	72.92 ± 12.42	-8.26 (-10.99, -5.54)	<0.001
	Not sure	69.07 ± 16.25	-11.70 (-16.40, -7.00)	<0.001
Sexual orientation	Homosexual	80.97 ± 11.10	Ref	
	Heterosexual	72.57 ± 12.83	-8.40 (-11.26, -5.53)	<0.001
	Bisexual	78.76 ± 14.37	-2.21 (-5.83, 1.42)	0.233
	Not sure	71.30 ± 15.61	-9.67 (-17.55, -1.79)	0.016
Type of sexual practice	Vaginal sex	72.58 ± 13.13	Ref	Ref
	Oral sex	73.83 ± 11.25	1.25 (-5.87, 8.37)	0.730
	Receptive anal sex	82.62 ± 15.31	10.04 (5.17, 14.91)	<0.001
	Insertive anal sex	82.41 ± 7.60	9.83 (5.49, 14.17)	<0.001
	Multiple sexual practices	80.28 ± 11.25	7.70 (4.86, 10.53)	<0.001
Multiple sexual partner	Yes	79.46 ± 11.41	Ref	Ref
	No	75.78 ± 13.87	-3.68 (-6.30, -1.06)	0.006
Age of sex debut (years)			-0.48 (-0.91, -0.05)	0.030
Condom use during 1^st^ sexual intercourse	Yes	78.15 ± 11.93	Ref	
	No	78.42 ± 16.96	0.27 (-3.52, 4.06)	0.887
	Non-disclosure	71.67 ± 12.84	-6.48 (-11.55, -1.42)	0.671
Condom use during last sexual intercourse	Yes	80.55 ± 10.07	Ref	
	No	72.20 ± 15.93	-8.36 (-11.38, -5.36)	<0.001
	Non-disclosure	69.71 ± 15.95	-10.84 (-15.41, -6.28)	<0.001
Type of visit to health clinic	New visit	80.00 ± 6.93	Ref	
	Follow up	77.69 ± 12.94	-2.31 (-15.06, 10.44)	0.722
Duration of HIV (years)			-0.43 (-0.67, -0.18)	<0.001
Duration of follow up (years)			-0.09 (-0.50, 0.32)	0.656
ARV treatment status	Yes	77.70 ± 12.61	Ref	
	No	87.50 ± 1.73	-6.63 (-9.69, -3.57)	0.122
Duration of ARV treatment			-0.45 (-0.73, -0.17)	0.002
Awareness of partners’ HIV status	Yes	79.28 ± 11.49	Ref	
	No	72.65 ± 15.29	-6.63 (-9.69, -3.57)	<0.001
Viral suppressed status (self-reported)	Yes	78.54 ± 11.76	Ref	
	No/unsure	74.23 ± 16.49	-4.32 (-7.63, -0.10)	0.011
Alcohol consumption	Yes	77.41 ± 10.91	Ref	
	No	78.01 ± 14.55	0.60 (-2.03, 3.23)	0.655
Illicit drug consumption	Yes	73.51 ± 11.01	Ref	
	No	79.39 ± 13.21	5.89 (3.03,8.74)	<0.001
Smoking status	Current/daily smoker	75.81 ± 11.76	Ref	
	Current/occasional smoker	86.08 ± 9.26	10.28 (2.41, 18.14)	0.345
	Nonsmoker	78.12 ± 13.83	2.31 (-0.74, 5.36)	0.137

Table 5 shows the multiple linear regression analysis for this study. Multicollinearity was detected between ‘sexual orientation’ and ‘type of sexual practice’, leading to the removal of ‘sexual orientation’ from the model. By using the stepwise method of linear regression, knowledge of STI (B = 0.73, p < 0.001), higher household income than RM10960 (B = 9.27, p < 0.001), last sexual activity three months or more ago (B = -4.34, p < 0.001), receptive anal sexual practice (B = 7.06, p < 0.001), and not using a condom during the last sexual intercourse (B = -4.43, p < 0.001) were significantly positively associated with condom use self-efficacy. The final model explained 30.7% of the variance in condom use self-efficacy (R² = 0.307).

**Table 5:** Multiple linear regression of the factors associated with condom use self-efficacy Multiple linear regression has been presented as Bb (95% CI). The p-value is considered statistically significant at p-value < 0.05. Dependent variable: condom use self-efficacy. Stepwise multiple linear method was applied. Model assumption was fulfilled. Multicollinearity was assessed. R2: 0.307. Bb: Adjusted regression coefficient, CI: Confidence interval, STI: Sexually transmitted infection

Variables		Multiple linear regression		
		Adjusted B^b^ (95% CI)	t	p-value
Total score of STI knowledge		0.73 (0.52,0.94)	0.336	<0.001
Last sexual activity	Less than 3 months	Ref		
	3 months or more	-4.34 (-6.77,-2.00)	-0.18	<0.001
	Not sure	1.60 (-4.63, 7.84)	-0.992	0.613
Household income	RM4850 – RM10959	Ref		
	RM4849 and below	-1.56 (-3.83, 2.49)	-0.670	0.503
	RM10960 and above	9.27 (4.69,13.85)	0.195	<0.001
Type of sexual practice	Vaginal sex	Ref		
	Oral sex	-5.82 (-12.81, 1.16)	-1.62	0.102
	Receptive anal sex	7.65 (2.95, 12.34)	3.21	<0.001
	Insertive anal sex	2.20 (-1.53, 5.93)	1.16	0.246
	Multiple sexual practices	1.54 (-10.64, 2.33)	0.47	0.637
Condom use during last sexual intercourse	Yes	Ref		
	No	-4.43 (-7.15,-1.72)	-0.163	<0.001
	Non-disclosure	-1.37 (-6.79, 3.21)	-0.62	0.533

## Discussion

The findings of our study indicate that the CUSES score among PLHIV in Hulu Langat, Selangor, is moderately high at 77.72 ± 12.88, corresponding to 72.0% of the maximum possible score. This suggests that while overall self-efficacy in condom use is relatively good, there is still room for improvement, particularly in areas identified by the subdomain scores. The "Mechanics" and "Assertiveness" subdomains showed high levels of confidence, indicating good practical skills and assertiveness in condom use. However, the "Perceived barrier" and "Intoxicant" subdomains revealed lower scores, highlighting potential challenges related to perceived obstacles and the impact of intoxicants on condom use efficacy. Comparing our results with other studies, it is evident that the self-efficacy scores can vary significantly across different populations and cultural contexts. For instance, Hamid et al. reported a slightly higher mean CUSES score of 82.93 ± 14.29 among youth attending RVD/STI clinics [12]. Conversely, Ahmad et al.'s research on men who have sex with men (MSM) in Malaysia found a lower mean CUSES score of 74.72 ± 19.33 [24]. Additionally, a recent study conducted in Brazil among PLHIV revealed a mean rank CUSES score of 50.00 in both male serodiscordant and seroconcordant PLHIV, emphasizing the importance of considering cultural and contextual factors in evaluating and addressing condom use self-efficacy [14].

STI knowledge and condom use self-efficacy

Our study revealed that the overall mean score of STI knowledge among PLHIV was 30.87 ± 5.50, representing 81.2% of the maximum possible score. This high score indicates a substantial level of awareness and understanding of STIs among the participants. These findings suggest that PLHIV has higher levels of STI knowledge compared to other studies in other populations in Malaysia [18,19]. Compared to other studies among the key population, our findings show a markedly higher level of STI knowledge. For instance, a study on female sex workers (FSW) revealed that only 51% had good STI knowledge [25]. Breaking down the STI knowledge into subdomains, we found that the "Risk factors of STI" and "Preventive measures of STI" subdomains had the highest mean scores, at 95.1% and 93.3% of the maximum scores, respectively. This suggests that participants are particularly knowledgeable about the factors that increase the risk of STI transmission and the measures that can be taken to prevent STIs. The "Transmission of STI" subdomain also showed a high level of knowledge, with a mean score of 6.46 ± 1.13, accounting for 92.2% of the maximum score. This indicates that the participants have a strong understanding of how STIs are transmitted, which is crucial for effective prevention. In contrast, the "Types of STI" subdomain had the lowest mean score at 6.73 ± 2.35, representing 67.3% of the maximum score. This lower score indicates that there is a gap in knowledge regarding the different types of STIs, which may affect the participant's ability to identify and respond to various infections appropriately. Similarly, the "Symptom of STI" subdomain had a mean score of 5.42 ± 1.82, or 67.7% of the maximum score, indicating a moderate level of awareness about the symptoms of STI.

Higher STI knowledge is significantly associated with higher condom use self-efficacy among PLHIV. This finding is consistent with other research but among a more general population. For example, Ma et al. found that better knowledge of STIs was positively associated with consistent condom use [25]. This is aligned with Bandura’s theory of self-efficacy. Better knowledge and awareness contribute to better self-efficacy through mastery experiences [15]. Successfully performing a task strengthens self-efficacy. The more individuals are exposed to knowledge and develop awareness through repeated successful experiences, the more their confidence in their abilities grows.

Household income

One of the key findings was that people living with HIV who have a household income of RM10960 or above tend to have higher condom use self-efficacy scores. This finding aligns with studies done by Li et al., which found that better income is associated with better condom use behavior [26]. The reason for this is that higher income provides economic stability and better affordability to purchase condoms. In a broader context, income has been shown to impact health behaviors and access to preventive measures, supporting our findings.

Receptive anal sexual practice

We also found that engaging in receptive anal sexual practices was significantly associated with higher condom use self-efficacy. PLHIV who practice receptive anal sex have a higher awareness of their increased risk for HIV and other STIs, as shown in previous studies done by Ye et al. and Wang et al. [27,28]. This perceived risk may motivate individuals to prioritize and feel more capable of using condoms.

Sexual activity and condom use behavior

Not using a condom during the last sexual intercourse was negatively associated with condom use self-efficacy. The failure to use a condom may reinforce a lack of confidence in one’s ability to use condoms, thereby lowering self-efficacy. Previous research indicates that individuals who do not use condoms consistently may have lower self-efficacy regarding their use [29]. This finding also aligns with Bandura’s theory, which suggests that past behavior influences self-efficacy beliefs [15].

Finally, our study found that being less sexually active is associated with lower condom use self-efficacy. Similar trends have been observed in the study by Aventin et al. in Southern Africa [30]. This finding also fits Bandura’s theory that past behavior influences self-efficacy. This could be because less sexually active individuals have less experience in practicing condom use, which, in turn, reduces their confidence in using condoms when they become sexually active. This finding underscores the need to promote confidence in condom use, even among those who are less sexually active, to ensure they are prepared to engage in safe sexual practices when necessary. The focus should be on providing support and education so that if individuals choose to engage in sexual activity, they can do so safely and confidently.

Limitations of the study

This study has several limitations. First, the cross-sectional design limits the ability to infer causality between the factors studied and condom use self-efficacy. Longitudinal studies would be beneficial in establishing causal relationships. Second, the use of self-reported data may introduce social desirability bias, potentially leading to over- or under-reporting of sexual behaviors and condom use. Third, the study was conducted in public health clinics within the Hulu Langat District, which may limit the generalizability of the findings to other regions or settings in Malaysia. Lastly, the convenience sampling method may not fully represent the broader population of PLHIV.

Implication for clinical practice and future research

Clinically, it is important to effectively increase condom use self-efficacy among PLHIV through enhancing STI knowledge and applying targeted interventions among those with lower income and those who are less sexually active. For populations with lower income, it is crucial to develop tailored educational programs that focus on safe sex practices and comprehensive STI knowledge. These programs should address the specific barriers to condom use. Utilizing community outreach and local resources can significantly enhance the dissemination and impact of this information. Reaching out to these communities will enable them better confidence to practice safer sex when required. 

Second, it is important not to overlook individuals who are less sexually active. Our study has shown that those who have not been sexually active PLHIV have lower condom use self-efficacy. While less sexual activity may reduce the risk of transmission, it is crucial to ensure that these individuals feel confident and prepared to use condoms if they choose to engage in sexual activity. Providing ongoing education and support can help build their confidence and preparedness, ensuring they can practice safe sex when the time comes.

Given that this research was conducted within a small setting of those attending HIV clinics in one district, it is essential to broaden the scope of future studies to include other PLHIV populations, especially among those in the community or defaulters. The expansion will help ensure that the findings are generalizable and applicable to a wider community. With proper sampling techniques, such as randomized sampling, it would be better to enhance the representativeness and reliability of the results. This approach will help mitigate any biases and improve the overall validity of the findings.

## Conclusions

The findings of this study provide valuable insights into the factors associated with condom use self-efficacy among people living with HIV (PLHIV) in Malaysia. The study highlights the significant role of STI knowledge, sexual activity, and demographic factors in influencing condom use self-efficacy. Despite the limitations, the results underscore the importance of sexual education and targeted interventions to enhance condom use self-efficacy, thereby promoting safer sexual practices among PLHIV.

## Appendices

Questionnaire 

**Table 6:** Eligibility criteria

I would like to ask you some questions about your sexual health and sexual practices. I understand that this question is very personal, but it is very important for the results of my study. The information you provide will be kept confidential. (Please circle your answer.)	
1. Are you 18 years old and older?	Yes/No
2. Can you read and understand Malay?	Yes/No
3. Do you have a mental health or psychiatric problem?	Yes/No

**Table 7:** Sociodemographic factors

Gender	Male	Female				Others/Transgender
Age	______________ years					
Race	Malay	Chinese		Indian		Others
Marital status	Single	Married				Widow/divorce
Educational status	No formal education Primary school (Standard 1-6) Secondary school (Form 1-5) Pre-university (STPM/Matriculation) Tertiary (Diploma/Degree/Master/PhD)					
Religion	Islam	Christian	Buddhist		Hindu	Others.
Occupation	Government sector Private sector Self-employed Housewife/unemployed Others					
Household income	RM4849 and below RM4850 – RM10959 RM10960 and above					

**Table 8:** Sexual behaviors

When was the last time you had sex?	Less than 3 months ago More than 3 months ago Not sure
What is your sexual orientation?	Heterosexual Homosexual Bisexual Not sure
What is your sexual practice? (You can tick more than one)	Vaginal sex Oral sex Anal sex bottom (receptive) Anal sex on the top (insertive) Multiple sexual practices
Have you had more than one sexual partner in the last 12 months?	Yes No
How old were you when you first had sex?	______________years old
Did you use a condom the first time you had sex?	Yes No Non-disclosure
Did you use a condom the last time you had sex?	Yes No Non-disclosure

**Table 9:** Clinical characteristics

Is this your first appointment or a follow-up appointment at the health clinic?	First appointment	Follow-up
If a follow-up appointment, how long has it been since you attended the follow-up appointment?	(_________________________) year	
How many years since you were first diagnosed with HIV?	(_________________________) year	
Are you currently taking medication to treat HIV?	Yes	No
If so, a) How long have you been taking the treatment?	(_________________________) year	
Do you know your partner's HIV status in the last 12 months?	Yes	No
Have you been told that you have a viral load of less than 20/not detected?	Yes	No
Have you ever consumed alcohol?	Yes	No
Have you ever taken illegal drugs?	Yes	No
Do you smoke?	Yes. Smoking every day Yes. Smoking occasionally Not smoking	

**Table 10:** Knowledge about sexually transmitted infection Please choose one answer either 'Yes', 'No' or 'Don't know' by marking/in the space or box provided for each answer

1. Which of the following is a sexually transmitted disease?	Answer					
	Yes			No		Don’t know
a. Syphilis						
b. Pneumonia						
c. HIV						
d. Tuberculosis						
e. Trichomoniasis						
f. Genital herpes						
g. Hepatitis B						
h. Gonorrhea						
i. Chlamydia						
j. Chickenpox						
2. Sexually transmitted diseases can generally be spread through:	Answer					
	Yes	No			Don’t know	
a. Vaginal sexual intercourse						
b. Anal intercourse						
c. Oral intercourse						
d. Using public toilets						
e. Blood transfusion						
f. From the pregnant mother (who is infected with sexually transmitted diseases) to the baby in the womb						
g. Shake hands with infected people						
3. In general, risk factors for sexually transmitted diseases are:	Answer					
	Yes		No		Don’t know	
a. Sexual activity that does not use a condom						
b. Multiple sexual partners						
c. Men who practice same-sex (MSM)						
d. Having sexual relations with sex workers						
e. Exposed to sexual activity at a young age						
f. Uncircumcised men						
g. Smoking						
4. In general, the signs and symptoms of sexually transmitted diseases are:	Answer					
	Yes		No		Don’t know	
a. Itching in the genital area						
b. Abnormal per vaginal discharge						
c. Vomiting						
d. Pain in the genital area during sexual activity						
e. Genital ulcer						
f. Headache						
g. Prolonged cough for more than 2 weeks						
h. No symptoms or signs						
5. In general, the way to control and prevent sexually transmitted diseases is through:	Answer					
	Yes	No			Don’t know	
a. Use of condoms						
b. Avoid sexual activity before marriage						
c. Be faithful to your partner						
d. Perform screening tests to detect sexually transmitted diseases						
e. Get early treatment if you are infected with a sexually transmitted disease						
f. Get vaccinated to prevent some sexually transmitted diseases						

**Table 11:** Condom use self-efficacy scale (CUSES)

The following questions are about how you feel about using a condom in certain situations. Please provide your response even if you have never used a condom (or have a partner who does). If you have never used it, please describe how you think when in that situation. Your answers will help us to plan programs in the future. Please circle ONE of your answer choices below. Please make sure all questions are answered.									
0		1	2	3	4				
Strongly Disagree		Disagree	Not sure	Agree	Strongly Agree				
	QUESTIONS								
	I feel confident in my ability to put a condom on myself or my partner.				0	1	2	3	4
	I feel confident I could remember to carry a condom with me should I need one.				0	1	2	3	4
	I feel confident in my ability to discuss condom usage with any partner I might have.				0	1	2	3	4
	I feel confident in my ability to suggest using condoms with a new partner.				0	1	2	3	4
	I feel confident I could suggest using a condom without my partner feeling ‘diseased’.				0	1	2	3	4
	I feel confident in my own or my partner's ability to maintain an erection while using a condom.				0	1	2	3	4
	I would feel embarrassed to put a condom on myself or my partner.				0	1	2	3	4
	If I were to suggest using a condom to a partner, I would feel afraid that he or she would reject me.				0	1	2	3	4
	If I were unsure of my partner’s feelings about using condoms, I would not suggest using one.				0	1	2	3	4
	I feel confident in my ability to use a condom correctly.				0	1	2	3	4
	I would feel comfortable discussing condom use with a potential sexual partner before we ever had any sexual contact (eg, hugging, kissing, caressing, etc).				0	1	2	3	4
	I feel confident in my ability to persuade a partner to accept using a condom after sexual intercourse.				0	1	2	3	4
	I feel confident I could gracefully remove and dispose of a condom after sexual intercourse.				0	1	2	3	4
	If my partner and I were to try to use a condom and did not succeed, I would feel embarrassed to try to use one again (eg, not being able to unroll the condom, putting it on backward, or awkwardness).				0	1	2	3	4
	I would not feel confident suggesting using condoms with a new partner because I would be afraid he or she would think I’ve had a past homosexual experience.				0	1	2	3	4
	I would not feel confident suggesting using condoms with a new partner because I would be afraid he or she would think I have a sexually transmitted disease.				0	1	2	3	4
	I would not feel confident suggesting using condoms with a new partner because I would be afraid he or she would think I thought they had a sexually transmitted disease.				0	1	2	3	4
	I would feel comfortable discussing condom use with a potential sexual partner before we ever engaged in intercourse.				0	1	2	3	4
	I feel confident in my ability to incorporate putting a condom on myself or my partner into foreplay.				0	1	2	3	4
	I feel confident that I could use a condom with a partner without ‘breaking’ the mood.				0	1	2	3	4
	I feel confident that 1 would remember to use a condom on myself or my partner quickly				0	1	2	3	4
	I feel confident I could use a condom during intercourse without reducing any sexual sensation.				0	1	2	3	4
	I feel confident I would remember to use a condom even after I have been drinking.				0	1	2	3	4
	I feel confident that I would remember to use a condom even if I were high.				0	1	2	3	4
	If my partner didn’t want to use a condom during intercourse, I could easily convince him or her that it was necessary to do so.				0	1	2	3	4
	I feel confident that I could use a condom successfully.				0	1	2	3	4
	I feel confident I could stop to put a condom on myself or my partner even in the heat of passion.				0	1	2	3	4
